# Nitrogen Topdressing Rate Alters Starch and Protein Properties in Grains at Different Spike Positions Under Long-Term Field Conditions

**DOI:** 10.3390/plants14233678

**Published:** 2025-12-03

**Authors:** Jiarui Wang, Haiyang Jin, Xiaoyan Zhang, Yonghui Hao, Baoting Fang, Deqi Zhang, Cheng Yang, Hanfang Wang, Junqin Yue, Hongjian Cheng, Fei Zheng, Xiangdong Li

**Affiliations:** 1 Wheat Research Institute, Henan Academy of Agricultural Sciences, Zhengzhou 450002, China; 2 College of Resources and Environmental Sciences, Henan Agricultural University, Zhengzhou 450046, China

**Keywords:** wheat, nitrogen topdressing, spike positions, starch accumulation, grain protein composition

## Abstract

Nitrogen (N) is a key nutrient influencing wheat growth, grain yield, and quality. A long-term field experiment was conducted using cultivar Zhengmai 1860 to clarify the effects of N topdressing on grain protein composition, starch accumulation, and yield. Treatments included a basal N application of 150 kg ha^−1^ (N1) combined with four topdressing rates at jointing: 37.5, 75, 112.5, and 150 kg ha^−1^ (N1 + 37.5, N1 + 75, N1 + 112.5, N1 + 150). Nitrogen topdressing significantly affected the physiological and biochemical characteristics of grains at different spike positions. Amylopectin, globulin, soluble starch (SS), and soluble starch synthase (SSS) accumulated most under 75–112.5 kg ha^−1^, with N1 + 75 showing the strongest response in basal and middle spike grains. Amylose and granule-bound starch synthase (GBSS) peaked at the middle spike under N1 + 112.5. Protein component (gliadin, glutelin, albumin), amino acids, glutamate synthase (GOGAT), and glutamine synthetase (GS) increased progressively with higher N rates, with maximum accumulation at N1 + 150. Nitrogen topdressing also enhanced spike number (5.05–37.13%), grains per spike (3.86–16.22%), and 1000-grain weight (2.72–5.79%), with the highest yield (9451.7 kg ha^−1^) at N1 + 112.5. These results highlight the critical role of optimized N management in improving grain composition and yield in wheat.

## 1. Introduction

Wheat (*Triticum aestivum* L.) is a staple cereal crop that contributes significantly to global food security, providing both calories and proteins for the human diet [1,2]. Achieving high yield and good quality simultaneously has become a central goal in wheat production [3], especially under the pressure of increasing population demand and limited arable land. Among agronomic practices, nitrogen (N) fertilization is one of the most critical factors influencing wheat growth, yield formation, and grain quality [4,5]. As a key nutrient, N strongly regulates the biosynthesis of starch and proteins in developing grains, thereby determining their nutritional and processing properties [6,7].

Excessive N applications are still common in agricultural production, as farmers often seek to maximize yields [8,9,10]. However, this practice frequently results in diminishing yield gains, decreased nitrogen use efficiency (NUE), and even yield decline at supra-optimal N levels, while also exacerbating environmental problems such as nitrate leaching and greenhouse gas emissions [11,12,13,14]. Previous studies have demonstrated that wheat yield and grain protein content typically follow a curvilinear response to N rates, increasing initially and then declining once an optimum is surpassed [15]. For instance, the yield-promoting effect of N is most significant at 200–250 kg ha^−1^, while the increase in grain protein content can be maximized at around 384 kg ha^−1^ [16]. The timing of nitrogen fertilizer application helps synchronize nitrogen supply with crop nitrogen demand. For example, topdressing nitrogen fertilizer at the jointing stage can significantly enhance the enzyme activity of primary nitrogen assimilation (e.g., glutamine synthetase (GS) and glutamate synthase (GOGAT) activities) [17], which promotes the production of glutamine and asparagine, providing ample precursor substances for the synthesis of glutenin and gliadin, while also supporting starch granule development by maintaining the carbon–nitrogen balance during the early grain filling stage, thereby increasing grain protein content, affecting starch structure characteristics, and ultimately determining grain quality [18,19,20,21]. As the main form of nitrogen storage, the effective addition of nitrogen fertilizer can significantly affect the grain protein content by increasing the activity of enzymes related to nitrogen metabolism, thereby changing the composition of grain components. It was found that excessive nitrogen shifts the metabolic allocation toward protein biosynthesis, increasing amino acid supply and proteosome formation. However, it diverts the carbon skeleton away from starch accumulation and inhibits the synthesis of soluble starch synthase (SSS) and granule-binding starch synthase (GBSS), thus reducing the number of starch granules and altering the ratio of proteosomes to starch granules [6,22,23,24]. These findings indicate that nitrogen fertilizer application exerts distinct regulatory effects on starch and protein biosynthesis and that optimizing nitrogen fertilizer input is crucial for balancing yield and quality [25,26].

Grain development within a wheat species exhibits a pronounced spatial-temporal sequence [27]. Grains located in central spikelets (superior grains) usually flower earlier, fill faster, and accumulate more assimilates compared with grains at basal or apical spikelets (inferior grains) [2,28]. Superior grains generally show a higher thousand-grain weight, starch content (amylose and amylopectin), protein content (gliadin and glutenin), enzymatic activity, and grain-filling rate [2,5,29,30], while inferior grains exhibit greater variability and are more sensitive to environmental and nutritional conditions [31,32]. Research finding: A delayed sowing date increased the distribution of ^13^C assimilates to spikes, especially to the upper and basal positions of spikes. Consequently, the number of grains in the upper position of the spike showed the greatest increase, followed by the basal and middle positions. A delayed sowing date also caused the most significant decrease in grain protein concentration at the basal of the spike, followed by the middle and upper positions [33]. It was found that both dry-hot wind and drought stress during the middle filling stage reduced the grain filling rate of inferior grains, resulting in a significant decrease in grain weight [34,35]. This grain positional effect has important implications for both yield formation and quality determination.

Although numerous studies have examined the effects of nitrogen fertilization on yield formation, grain quality, and nitrogen use efficiency, most have focused on whole-spike responses and short-term trials. To address these existing knowledge gaps, we conducted a long-term field experiment with the wheat cultivar Zhengmai 1860 to investigate how nitrogen topdressing at the jointing stage regulates grain development. Studies explicitly integrating nitrogen regulation with positional differences among superior and inferior grains remain limited, particularly under long-term, production-scale field conditions. Based on the above research status, we conducted a long-term field positioning experiment with different N topdressing rates during the wheat jointing stage. By investigating yield components and the differences in starch and protein accumulation, amino acid content, and the activities of enzymes involved in N and C among grains at different spike positions, this study aims to (i) clarify how N regulates starch and protein synthesis in grains at different spike positions and (ii) elucidate the physiological mechanisms underlying the differential responses of grains to N supply. The innovation of this study lies in integrating different N topdressing rates with grain spatial heterogeneity, thereby enabling in-depth analysis of the physiological mechanisms underlying the changes in grain starch and protein driven by N fertilizer. The research results provide theoretical support for precise N management strategies in wheat production, thereby achieving the simultaneous improvement of yield and grain quality.

## 2. Results

### 2.1. Effects of Nitrogen Topdressing Rates on Soluble Sugar Content in Grains at Differential Spike Positions

**Figure 1:**
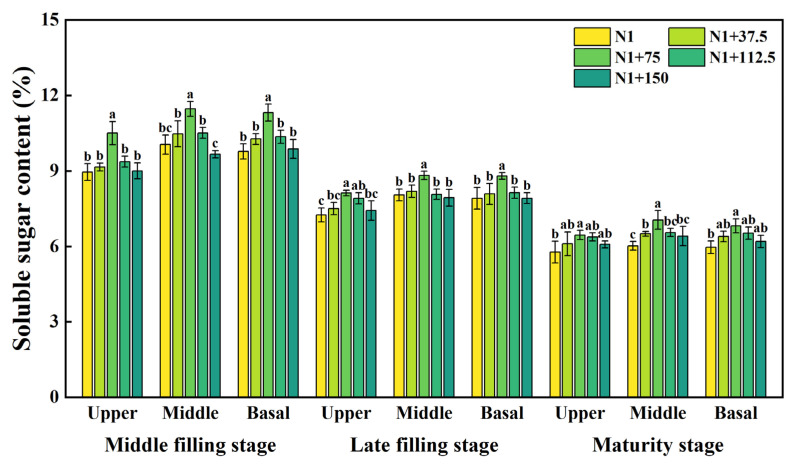
Effects of different nitrogen topdressing rates on the soluble sugar content in grains at different spike positions. Different lowercase letters in the same group indicate significant differences among treatments at *p* < 0.05. Abbreviations: N1—Basal nitrogen application of 150 kg ha^−1^; N1 + 37, N1 + 75, N1 + 112.5, N1 + 150—Based on the basal application of nitrogen fertilizer 150 kg ha^−1^ and topdressing at the jointing stage of 37.5, 75, 112.5, and 150 kg ha^−1^, respectively.

The soluble sugar content showed obvious dynamic changes with the advancement of the growth period (Figure 1), with the highest content in the middle filling stage. It is indicated that the process of sugar synthesis and transport is most active at this time and sugar accumulation is at its peak. The effects of nitrogen topdressing rates on soluble sugar content were significantly different at each period. At the middle filling stage, the soluble sugar content of differential spike positions was significantly affected by different nitrogen topdressing rates, which was highest in the N1 + 75 treatment. With the increasing nitrogen topdressing rates, the soluble sugar content in the middle of the spike showed a trend of increasing and then decreasing. Compared with N1 treatment, N1 + 37.5, N1 + 75, and N1 + 112.5 increased by 4.29%, 14.13%, and 4.61%, respectively, while N1 + 150 decreased by 3.78%; both the upper and basal position of the spike were significantly different under N1 + 75 treatment and the soluble sugar content in the middle position of the spike was significantly higher than that in the upper and basal position of the spike. At the late filling stage, each treatment had a significant effect on the soluble sugar content in the upper position of the spike, which increased by 3.43%, 12.05%, 9.13%, and 2.40%, respectively, compared with N1, and the contents in the middle and basal position of the spike significantly increased by 9.62% and 8.82% under the N1 + 75 treatment, respectively. At maturity, soluble sugar contents in the upper, middle, and basal position of the spike were all significantly increased under N1 + 75 treatment, by 5.74%, 8.46%, and 6.58%, respectively, compared with N1. The soluble sugar contents of the upper, middle, and basal position of the spike were significantly different in all growth periods, with the middle position of the spike being significantly higher than the contents of the upper and basal position of the spike, indicating that sugar synthesis and translocation were more active in the middle position of the spike.

### 2.2. Effects of Nitrogen Topdressing Rates on the Enzyme Activity of GBSS in Grains at Differential Spike Positions

**Figure 2:**
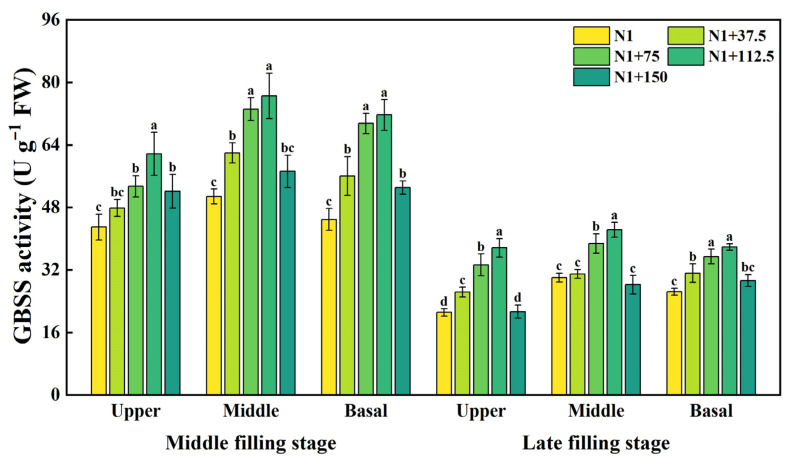
Effects of different nitrogen topdressing rates on GBSS enzyme activity in grains at different spike positions. Different lowercase letters in the same group indicate significant differences among treatments at *p* < 0.05. Abbreviations—N1: Basal nitrogen application of 150 kg ha^−1^; N1 + 37, N1 + 75, N1 + 112.5, N1 + 150—Based on the basal application of nitrogen fertilizer 150 kg ha^−1^ and topdressing at the jointing stage of 37.5, 75, 112.5, and 150 kg ha^−1^, respectively.

GBSS enzyme activities differed significantly between fertility periods and treatments (Figure 2). GBSS activity was higher at the middle filling stage; GBSS activity at the upper, middle, and basal position of the spike showed a trend of increasing and then decreasing with the increase in the nitrogen topdressing rates: N1 + 37.5, N1 + 75, N1 + 112.5, and N1 + 150 increased by 11.34%, 24.30%, 43.59%, and 21.28%, respectively, in the upper position of the spike, 22.01%, 44.08%, 50.72%, and 12.71%, respectively, in the middle position of the spike, and 24.80%, 54.72%, 59.59%, and 18.12% at the basal of the spike, respectively, compared with N1. N1 + 75 and N1 + 112.5 showed higher GBSS activity under different nitrogen topdressing rates, which may be related to the nitrogen supply promoting starch synthase activity. In addition, GBSS activity was significantly higher in the middle position of the spike than in the upper and lower position of the spike, indicating that starch synthesis was more active in the middle position of the spike. At the late filling stage, the overall GBSS activity decreased significantly but still maintained a high level, and the treatments N1 + 75 and N1 + 112.5 with higher nitrogen topdressing rates still had a significant effect on GBSS activity. Compared with N1, the upper position of the spike increased by 57.68% and 78.22%, the middle position of the spike increased by 28.98% and 40.86%, and the basal of the spike increased by 34.05% and 43.41%, respectively, meaning that the appropriate amount of nitrogen can significantly promote the GBSS activity and the upper position of the spike in the later stages of reproduction has a larger space for starch synthesis.

### 2.3. Effects of Nitrogen Topdressing Rates on the Enzyme Activity of SSS in Grains at Differential Spike Positions

**Figure 3:**
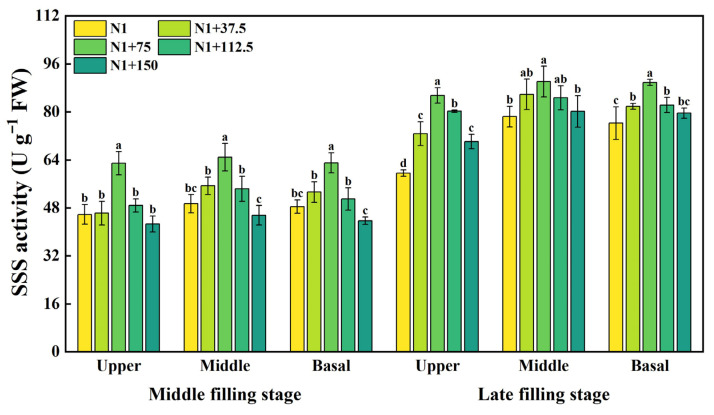
Effects of different nitrogen topdressing rates on SSS enzyme activity in grains at different spike positions. Different lowercase letters in the same group indicate significant differences among treatments at *p* < 0.05. Abbreviations—N1: Basal nitrogen application of 150 kg ha^−1^; N1 + 37, N1 + 75, N1 + 112.5, N1 + 150—Based on the basal application of nitrogen fertilizer 150 kg ha^−1^ and topdressing at the jointing stage of 37.5, 75, 112.5, and 150 kg ha^−1^, respectively.

The SSS activity showed an overall trend of increasing and then decreasing with the increase in the nitrogen topdressing rates (Figure 3), and the response to it varied among different spike positions. In the mid-filling stage, SSS activity in the upper, middle, and basal positions of the spike was significantly affected in the N1 + 75 treatment, which increased by 37.32%, 31.44%, and 30.16%, respectively, while it decreased significantly in the N1 + 150 treatment, decreasing by 6.89%, 7.80%, and 9.72%, respectively, compared with N1, with the middle and basal positions of the spike reaching a significant difference, indicating that the appropriate nitrogen topdressing rates positively regulated SSS activity. With the advancement of the growth period, SSS activity significantly increased in the late filling stage and showed a trend of initially increasing and then decreasing with the increase in nitrogen topdressing rates; the activities of the upper, middle, and basal of the spike reached the highest level under the N1 + 75 treatment, which increased by 43.42%, 14.89%, and 17.78%, respectively, compared with N1, and the upper position of the spike had a higher enzyme activity, indicating that its starch synthesis capacity was stronger, which promoted starch granule rapid accumulation.

### 2.4. Effects of Nitrogen Topdressing Rates on the Amylose Content in Grains at Differential Spike Positions

**Figure 4:**
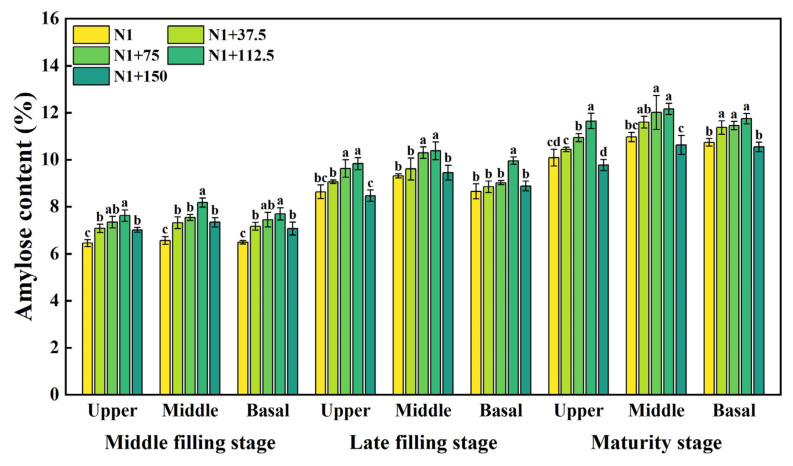
Effects of different nitrogen topdressing rates on amylose content in grains at different spike positions. Different lowercase letters in the same group indicate significant differences among treatments at *p* < 0.05. Abbreviations—N1: Basal nitrogen application of 150 kg ha^−1^; N1 + 37, N1 + 75, N1 + 112.5, N1 + 150—Based on the basal application of nitrogen fertilizer 150 kg ha^−1^ and topdressing at the jointing stage of 37.5, 75, 112.5, and 150 kg ha^−1^, respectively.

The effect of nitrogen topdressing rates on the amylose content at all spike positions was significantly different at each period. The amylose content in the grains of each spike showed a gradually increasing trend of change (Figure 4). At the mid-filling stage, the amylose content in the upper, middle, and basal positions of the spike showed an increasing and then decreasing trend with the increase in nitrogen topdressing rates, which had a significant effect under the N1 + 112.5 treatment, as the amylose content increased by 18.15%, 24.55%, and 18.61%, respectively, and there was higher accumulation in the middle part of the spike compared to that of N1, while the other treatments of nitrogen topdressing rates did not reach a significant level. At the late filling stage, the amylose content in each spike position also showed a trend of increasing and then decreasing, in which the upper and middle positions of the spike increased significantly under the N1 + 75 and N1 + 112.5 treatments, and compared with N1, the upper position of the spike increased by 11.48% and 13.85%, and the middle position of the spike increased by 10.56% and 11.40%, respectively; the base of the spike only reached a significant level under the N1 + 112.5 treatment. The trend of changes in the amylose content at maturity was consistent with the performance at the filling stage, showing a single peaked curve, with the content in the upper, middle, and basal positions of the spike being the highest under the N1 + 112.5 treatment, increasing by 15.54%, 10.90%, and 9.42%, respectively, as compared to N1; the content was reduced under the N1 + 150 treatment but did not reach a significant level.

### 2.5. Effects of Nitrogen Topdressing Rates on the Amylopectin Content in Grains at Differential Spike Positions

**Figure 5:**
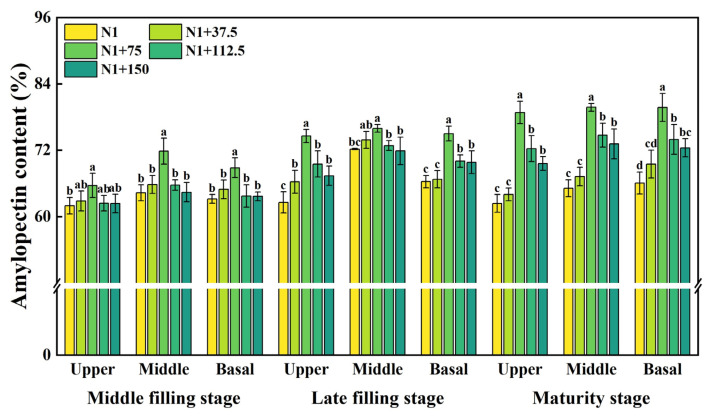
Effects of different nitrogen topdressing rates on amylopectin content in grains at different spike positions. Different lowercase letters in the same group indicate significant differences among treatments at *p* < 0.05. Abbreviations—N1: Basal nitrogen application of 150 kg ha^−1^; N1 + 37, N1 + 75, N1 + 112.5, N1 + 150—Based on the basal application of nitrogen fertilizer 150 kg ha^−1^ and topdressing at the jointing stage of 37.5, 75, 112.5, and 150 kg ha^−1^, respectively.

The effect of nitrogen topdressing rates on the amylopectin content of all spike positions was found in all periods, and the change in the content was not significant with the advancement of growth. The amylopectin content at the upper, middle, and basal positions of the spike in all periods showed an increasing and then decreasing trend with the nitrogen topdressing rates (Figure 5). There was a significant effect on the content under the N1 + 75 treatment, which increased by 5.91%, 11.68%, and 8.92% in the mid-filling stage and by 19.20%, 5.18%, and 13.09% in the late filling stage, respectively, compared to N1, with higher amylopectin content in the middle position of the spike. There was an overall decrease in the content of all spike positions at maturity. Nitrogen topdressing rates significantly increased the amylopectin content, which was significantly higher under the N1 + 75 treatment, increasing by 26.35%, 22.50%, and 20.70%, respectively, as compared to N1, and also under the N1 + 112.5 and N1 + 150 treatments, where it reached a significant level.

### 2.6. Effects of Nitrogen Topdressing Rates on the Amino Acid Content in Grains at Differential Spike Positions

**Figure 6:**
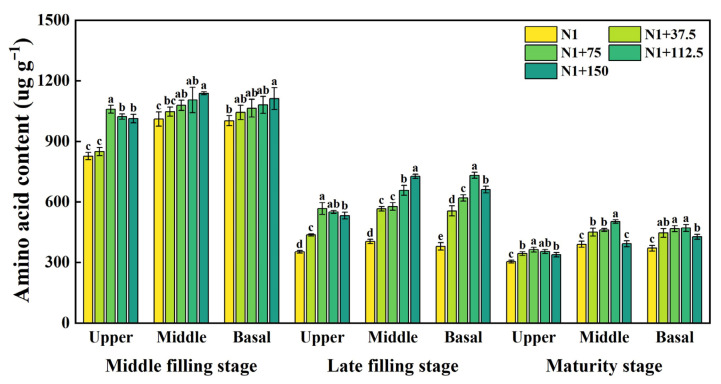
Effects of different nitrogen topdressing rates on amino acid content in grains at different spike positions. Different lowercase letters in the same group indicate significant differences among treatments at *p* < 0.05. Abbreviations—N1: Basal nitrogen application of 150 kg ha^−1^; N1 + 37, N1 + 75, N1 + 112.5, N1 + 150—Based on the basal application of nitrogen fertilizer 150 kg ha^−1^ and topdressing at the jointing stage of 37.5, 75, 112.5, and 150 kg ha^−1^, respectively.

The free amino acid content of the grains in each spike position showed a gradual decrease as fertility progressed, with a larger decrease from the mid to late filling stage (Figure 6). The free amino acid content in the middle and basal positions of the spike at the mid-filling stage and in the middle position of the spike at the late filling stage showed a gradual increase with the increase in nitrogen topdressing rates at the jointing stage, while the free amino acid content in each spike position at all other periods showed a trend of increasing and then decreasing with nitrogen topdressing rates. At the mid-filling stage, the free amino acid content of the upper position of the spike differed significantly under N1 + 75, N1 + 112.5, and N1 + 150, increasing by 28.15%, 23.74%, and 22.57%, respectively, compared with N1. In the late filling and maturity stage, the amino acid content of the upper and base position of the spike differed significantly with each nitrogen topdressing rate, with free amino acid content in the middle position of the spike at the maturity stage being the highest.

### 2.7. Effects of Nitrogen Topdressing Rates on the GOGAT Activity in Grains at Differential Spike Positions

**Figure 7:**
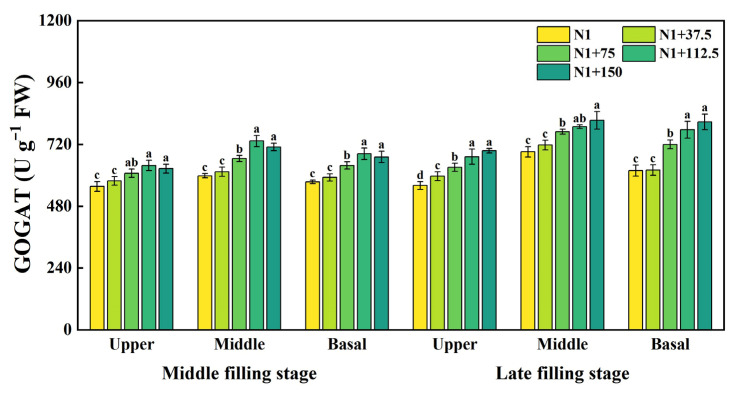
Effects of different nitrogen topdressing rates on GOGAT enzyme activity in grains at different spike positions. Different lowercase letters in the same group indicate significant differences among treatments at *p* < 0.05. Abbreviations: N1—Basal nitrogen application of 150 kg ha^−1^; N1 + 37, N1 + 75, N1 + 112.5, N1 + 150—Based on the basal application of nitrogen fertilizer 150 kg ha^−1^ and topdressing at the jointing stage of 37.5, 75, 112.5, and 150 kg ha^−1^, respectively.

Grain glutamate synthase GOGAT activity at the top, middle, and basal of the spike at the mid-filling stage showed an increasing and then decreasing trend with increasing nitrogen topdressing rates (Figure 7). There was a significant increase in GOGAT activity under the N1 + 75, N1 + 112.5, and N1 + 150 treatments, with the highest activity under the N1 + 112.5 treatment, which increased by 14.70%, 22.56%, and 18.98% at each spike position, respectively, compared to N1. With the advancement of growth, overall enzyme activity increased at the late filling stage, and GOGAT activity in the upper, middle, and basal positions of the spike all increased with increasing nitrogen topdressing rates. GOGAT activity reached significant levels under N1 + 75, N1 + 112.5, and N1 + 150 treatments, and the enzyme activity in the upper position of the spike increased by 12.64, 19.97, and 24.02%, respectively, as compared to N1. The enzyme activity of the middle position of the spike was elevated by 11.32%, 14.13%, and 17.72% and was elevated by 16.44%, 25.63%, and 30.65%, respectively, in the basal position of the spike, which was higher than that at the middle position of the spike, and the higher enzyme activity was favorable for driving the synthesis of grain proteins.

### 2.8. Effects of Nitrogen Topdressing Rates on the GS Activity in Grains at Differential Spike Positions

**Figure 8:**
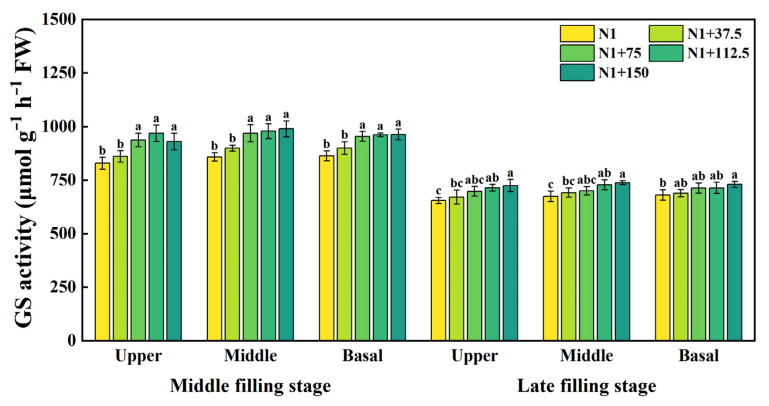
Effects of different nitrogen topdressing rates on GS enzyme activity in grains at different spike positions. Different lowercase letters in the same group indicate significant differences among treatments at *p* < 0.05. Abbreviations: N1—Basal nitrogen application of 150 kg ha^−1^; N1 + 37, N1 + 75, N1 + 112.5, N1 + 150—Based on the basal application of nitrogen fertilizer 150 kg ha^−1^ and topdressing at the jointing stage of 37.5, 75, 112.5, and 150 kg ha^−1^, respectively.

With the advancement of growth, GS enzyme activity decreased significantly at the late filling stage (Figure 8). At the mid-filling stage, the GS activity in the upper position of the spike showed an increasing and then decreasing trend with the increase in nitrogen topdressing rates, increasing significantly in the range of 75–150 kg ha^−1^, with the highest increase at 112.5 kg ha^−1^. The enzyme activities in the middle position of the spike and the basal position of the spike also increased significantly in the range of 75–150 kg ha^−1^ but the differences between treatments were not significant. In the late filling stage, the GS activity increased with the increase in nitrogen topdressing rates, and the GS activity at the upper, middle, and basal of the spike was the highest under the N1 + 150 treatment, increasing by 10.68%, 9.44%, and 7.23%, respectively, compared with N1. Adequate nitrogen fertilizer supply accelerates nitrogen assimilation by upregulating GS enzyme activity to promote grain development and protein accumulation.

### 2.9. Effects of Nitrogen Topdressing Rates on the Protein Component Content in Grains at Differential Spike Positions

**Figure 9:**
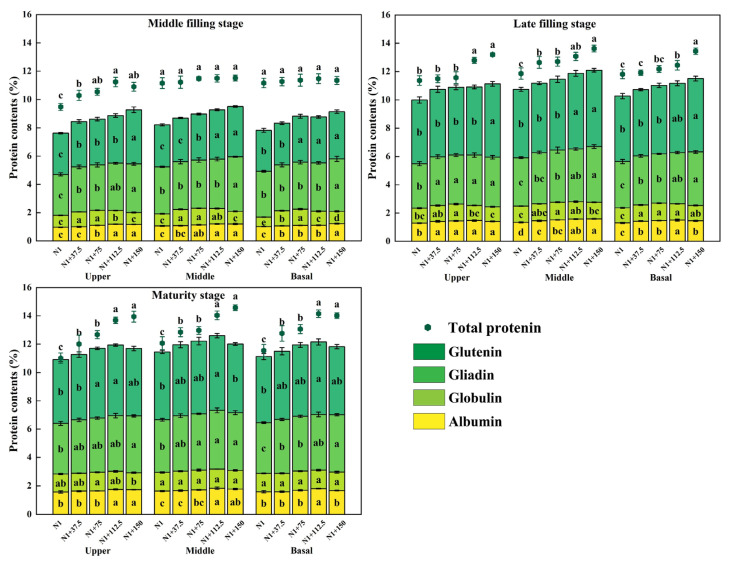
Effects of different nitrogen topdressing rates on protein component content in grains at different spike positions. Within the same group and color category, different lowercase letters indicate significant differences among treatments at *p* < 0.05. Each plot has five components, with the scatter plot at the top of the bar representing the change in total protein content and the bars from top to bottom showing glutenin, gliadin, globulin, and albumin content. Abbreviations: N1—Basal nitrogen application of 150 kg ha^−1^; N1 + 37, N1 + 75, N1 + 112.5, N1 + 150—Based on the basal application of nitrogen fertilizer 150 kg ha^−1^ and topdressing at the jointing stage of 37.5, 75, 112.5, and 150 kg ha^−1^, respectively.

Compared with the mid-filling stage, the contents of storage proteins (gliadin and glutenin) in the upper, middle, and basal grains at the maturity stage increased significantly, accounting for 62.91–73.48%, 61.26–70.52%, and 63.24–71.49% of the total protein content, respectively (Figure 9). It was also found that the protein component content in each spike position was mainly affected by the nitrogen topdressing rates, and the increase in nitrogen topdressing rates enhanced gliadin and glutenin content more than albumin and globulin. The albumin content increased first and then decreased with the increase in nitrogen topdressing in the upper and middle positions of the spike at the mid-filling stage, where the content was the highest under the N1 + 112.5 treatment but higher under the N1 + 150 treatment in the basal position of the spike. The globulin content decreased gradually with the increase in nitrogen topdressing in the mid-filling stage, late-filling stage, and maturity stage, being higher in the range of 37.5–75 kg ha^−1^ nitrogen topdressing. The gliadin content at the middle and late filling stages increased with the increase in nitrogen topdressing (middle and basal positions of spike); the glutelin content showed the same trend, which was the highest under the N1 + 150 treatment, and the difference was significant. At the mature stage, the glutelin content showed a decrease with the increase in nitrogen topdressing rate, and it was the highest under the N1 + 112.5 treatment. Compared with N1, the content of the upper, middle, and basal positions of spike increased by 10.27%, 10.11%, and 9.47%, respectively.

### 2.10. Effects of Different Nitrogen Topdressing Rates on Grain Yield and Yield Components

As can be seen from Table 1, there was a significant effect on the spike number, grain number per spike, 1000-grain weight, and yield with the increase in nitrogen topdressing rates. Compared with N1, all nitrogen topdressing rate treatments significantly increased the spike number by 5.05–37.13%, the grain number per spike by 3.86–16.22%, and the 1000-grain weight increased by 2.72–5.79% in the range of 37.5–112.5 kg ha^−1^ nitrogen topdressing rates and decreased in the 150 kg ha^−1^ treatment. Compared to N1, the N1 + 37.5, N1 + 75, N1 + 112.5, and N1 + 150 treatments showed an increase in yield of 9.15%, 25.13%, 28.46%, and 9.77%, respectively, which was highest under the 112.5 kg ha^−1^ treatment at 9451.74 kg ha^−1^.

### 2.11. Cluster Analysis of Physiological and Biochemical Traits of Wheat Grain

**Figure 10:**
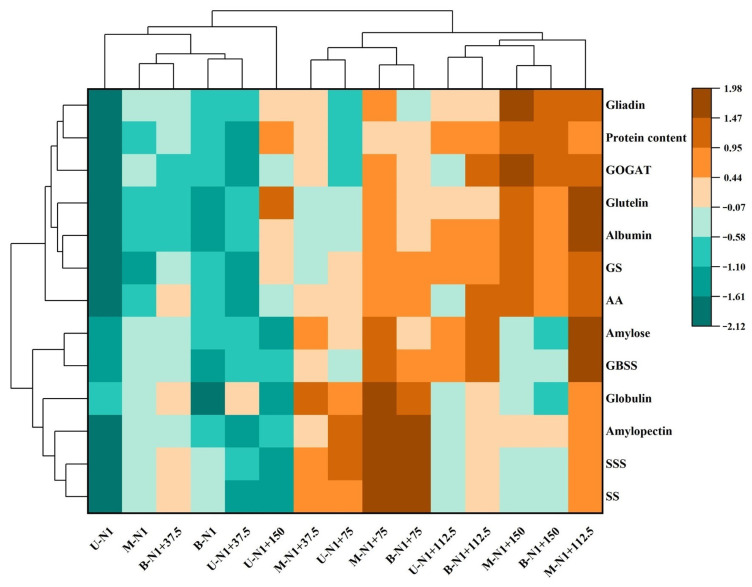
Cluster heatmap of grain physiological and biochemical indicators. U: Upper, M: Middle, B: Basal. Abbreviations: N1—Basal nitrogen application of 150 kg ha^−1^; N1 + 37, N1 + 75, N1 + 112.5, N1 + 150—Based on the basal application of nitrogen fertilizer 150 kg ha^−1^ and topdressing at the jointing stage of 37.5, 75, 112.5, and 150 kg ha^−1^, respectively.

The physiological and biochemical indexes of grains under different nitrogen topdressings and different spike positions were analyzed by cluster heat map (Figure 10). The change in the heat map color automatically reflected the magnitude and difference in data. According to the clustering results, there were significant differences in grain physiological indexes among different nitrogen topdressing rates and spike positions. SS, SSS, amylopectin, and globulin were clustered into one group, and these four indicators showed a decrease with the increase in nitrogen topdressing, and accumulation was higher within the range of 75–112.5 kg ha^−1^ of nitrogen topdressing, with the highest accumulations in the middle and basal positions of the spike in the N1 + 75 treatment. The performance of amylose and GBSS was consistent, and accumulation in the middle position of the spike was highest under the N1 + 112.5 treatment. Gliadin, protein content, GOGAT, glutelin, albumin, GS, and AA decreased with the increase in nitrogen topdressing rate and were highest under the N1 + 112.5 and N1 + 150 treatments in the middle position of spike.

## 3. Discussion

### 3.1. Effects of Nitrogen Management on Sugar Content and Starch Properties in Grains at Different Spike Positions

Nitrogen management critically regulates carbon assimilation and allocation during wheat grain filling, thereby influencing sugar availability for starch biosynthesis [6,36,37]. In this study, soluble sugar content responded positively to moderate nitrogen topdressing (75–112.5 kg ha^−1^) but declined at higher rates (Figure 1), suggesting that excessive nitrogen disrupts the carbon–nitrogen balance and reduces assimilate supply to grains [38,39]. As grain filling progressed, sugar content decreased continuously, reflecting senescence-induced reductions in photosynthesis and sugar synthase activity in flag leaves [40,41,42,43]. Importantly, spatial heterogeneity was evident: grains in the middle spikelet consistently accumulated more soluble sugars than those at basal or upper positions, which was mainly related to spikelet differentiation, as shown by Zheng et al. [44]; during spike development in wheat, the middle spikelets differentiate first, followed by the upper and basal spikelets. Thus, the middle spikelet has the “master advantage” and a stronger ability to acquire nutrients, while the upper and basal spikelets lag behind. The upper and basal spikelets have a relatively weak ability to acquire nutrients because of their delayed differentiation [45]. M. Reynolds et al. [46] also found that when the supply of assimilates is restricted, the middle spikelet can obtain a greater assimilate supply because its development occurs earlier and is thus more complete, while the upper and basal spikelets are more restricted regarding the supply of assimilates due to later development. This spatial pattern persisted through late filling and maturity, indicating that assimilate partitioning among grains is highly coordinated with spike developmental order [47,48]. Together, these results highlight that moderate nitrogen supply not only sustains sugar availability but also optimizes its spatial distribution, favoring starch accumulation particularly in the middle spikelet.

The biosynthesis of starch in the plant endosperm is regulated by a series of enzymes [49]. After phosphorylation on the surface of starch granules, GBSSI regulates the synthesis of amylose in the form of oligomers [50], while SSS and SBE are responsible for the synthesis of amylopectin [49,51,52]. Soluble sugars serve as substrates for starch synthesis, and their content is closely correlated with starch accumulation. Our results showed that the increase in nitrogen application rate could increase the GSSS and SSS activity, amylopectin, and amylose content of superior and inferior grains (Figure 2, Figure 3, Figure 4 and Figure 5). Within the nitrogen range of 75–112.5 kg ha^−1^, these parameters exhibited a linear increase with nitrogen topdressing rates. However, when the additional nitrogen reached 150 kg ha^−1^, these characteristics were inhibited. The observed decline was associated with reduced activities of GBSS and SSS during the middle and late grain filling stages, along with downregulated expression of AGPase, GBSSI, and GBSSII under high nitrogen conditions [21]. Some studies have also confirmed that nitrogen application at 300 kg ha^−1^ leads to a decrease in starch synthase activity, while application rates between 100 and 200 kg ha^−1^ enhance it [53]. Such patterns agree with earlier reports that appropriate nitrogen fertilization facilitates the translocation of dry matter stored in vegetative organs to grains, positively influencing grain sucrose content by increasing substrate availability for starch synthesis [54]. Conversely, excessive nitrogen application may produce opposite effects, ultimately inhibiting related enzymatic activities [18,39,55,56].

Moreover, the effects of nitrogen on enzyme activity and starch composition were more pronounced in basal and upper spikelets than in middle ones, suggesting that inferior grains are more sensitive to fluctuations in source-sink regulation under varying nitrogen supply [31]. These findings indicate that optimal nitrogen management not only supports sugar availability but also maintains enzyme activity for starch biosynthesis, thereby improving the filling of inferior grains and enhancing overall yield formation.

### 3.2. Effects of Nitrogen Management on Nitrogen Metabolism and Protein Components in Grains at Different Spike Positions

Nitrogen topdressing significantly increased free amino acid content in wheat grains, with the strongest effects observed in the upper and basal spikelet. As nitrogen rates increased from 75 to 150 kg ha^−1^, differences in amino acid accumulation among spike positions decreased (Figure 6), indicating that moderate N supply improves nitrogen distribution within the spike and mitigates developmental asynchrony among grains. This response is likely driven by enhanced nitrogen assimilation and mobilization, as topdressing stimulates the activity of key enzymes in the primary N assimilation pathway, including glutamine synthetase (GS) and glutamate synthase (GOGAT) [18,57]. During mid-to-late grain filling, the observed increase in GOGAT activity, particularly in basal grains (Figure 7), suggests that these grains possess a higher capacity for N translocation and utilization under sufficient N supply, which in turn supports the elevated synthesis of amino acids. Such effects reflect the intimate coupling between nitrogen and carbon metabolism, where the tricarboxylic acid (TCA) cycle provides carbon skeletons and energy for amino acid biosynthesis [58], while amino acids and enzymes from N metabolism reciprocally support carbon assimilation and carbohydrate partitioning [59]. Together, these processes explain why inferior grains are particularly responsive to nitrogen topdressing and how appropriate N management can synchronize grain development by improving N assimilation efficiency.

Beyond its influence on total N assimilation, N management also modified protein composition, although the magnitude and pattern of change varied among different protein fractions, which consistent with previous reports [60,61]. N topdressing reduced the gliadin-to-glutelin ratio, while promoting glutelin accumulation, which is favorable for improving the nutritional quality of rice grains [62]. The results of this study showed that with the increase in nitrogen topdressing, the response of grain protein content to nitrogen fertilizer was different, and the increase in protein content in different grain positions was different. The contents of albumin and gliadin in grains of different ear positions at the maturity stage were significantly increased at 112.5–150 kg ha^−1^ nitrogen topdressing, but the difference between spike positions was not significant, while the response of glutenin to nitrogen was the most significant at 112.5 kg ha^−1^ nitrogen topdressing (Figure 9). Globulin content, in contrast, was relatively insensitive to N application.

These observations indicate that suitable N topdressing not only increases total protein content but also modulates the relative proportions of protein fractions, with the regulatory effect being more pronounced in grains that are typically considered inferior. This aligns with the concept that targeted N management can enhance the sink capacity and protein biosynthetic potential of inferior grains, thereby contributing to both grain yield stability and end-use quality [63,64]. The differential response of spike positions to N application may be attributed to variations in source-sink dynamics, phloem transport capacity, and enzymatic activity among spikelets [1,28]. Upper and basal grains, which often experience N limitation due to positional effects, appear to benefit more from supplemental N, likely because topdressing enhances remobilization of pre-stored N and supports localized enzymatic activity for amino acid synthesis [30,65,66]. Moreover, changes in protein fraction composition may reflect preferential allocation of N to functionally important proteins, such as glutelin and glutenin, which are critical for dough quality [67,68]. Collectively, these findings emphasize that precision N management not only boosts overall grain protein content but also fine-tunes the nutritional and functional quality of wheat, particularly by narrowing developmental disparities among spikelets. In this study, the grain yield also increased significantly with the increase in nitrogen topdressing rate. Under 37.5–112.5 kg ha^−1^ nitrogen topdressing, spike number, grain number per spike, and 1000-grain weight increased significantly, and the yield increased by 9.15–28.46%. However, under 75 and 112.5 kg ha^−1^ nitrogen topdressing, there was no significant difference in yield. With the increase in nitrogen topdressing (150 kg ha^−1^), compared with N1, the grain yield increased by only 9.77%, and the 1000-grain weight decreased. The increase in yield was mainly through the increase in spike number and grain number per spike. Appropriate amounts of nitrogen topdressing can significantly improve the economic benefits of grain yield. Optimizing both the timing and rate of N application, therefore, represents a practical strategy for synchronizing grain development, improving NUE, and achieving simultaneous gains in yield and quality.

## 4. Materials and Methods

### 4.1. Plant Materials and Experimental Design

The long-term positioning experiment began in October 2015, located in Henan Modern Agricultural Research and Development Base (35°00′ N, 113°41′ E). The area is characterized by a warm-temperate zone in a semi-moist continental monsoon climate. The mean annual precipitation is approximately 600–800 mm, mostly falling between July and August, and the average annual temperature is 14.1 °C. The agrometeorological data were obtained from the meteorological data acquisition system near the experimental area (25 m) (Zhejiang top yunnong Technology Co., Ltd., Hangzhou, China, TP-WMS-1P). The soil type is tidal soil, with 0–20 cm soil organic carbon content of 4.29 g kg^−1^, total nitrogen of 0.31 g kg^−1^, total phosphorus of 0.87 g kg^−1^, total potassium of 19.65 g kg^−1^, and a pH value of 8.52.

The experiment was a randomized block design with five treatments in 2021–2022. The basal application of urea (N 46%) was 150 kg ha^−1^ (N1); based on basal application, the following urea (N 46%) was applied at the jointing stage: 37.5 kg ha^−1^ (N1 + 37.5), 75 kg ha^−1^ (N1 + 75), 112.5 kg ha^−1^ (N1 + 112.5), and 150 kg ha^−1^ (N1 + 150). Each treatment had three replicates, a total of 15 plots. The plot area was 6 m × 5 m, separated by concrete with a depth of 2 m. The basal application of urea (N 46%), calcium superphosphate (P_2_O_5_, 12%) 120 kg ha^−1^, and potassium chloride (K_2_O, 60%) 90 kg ha^−1^ were evenly broadcast to the ground before sowing and then incorporated into the soil by rotary tillage (at a depth of 0–20 cm). Zhengmai 1860, a large-scale wheat variety in the Huanghuai wheat area, was used as the test material and sown on October 24, 2021. Field management measures were consistent in all the plots except for nitrogen fertilizer.

### 4.2. Sampling and Measurements

During the wheat flowering period, plants that exhibited uniform growth and flowered on the same day were carefully chosen and tagged for further experimentation, sampled on May 11 (mid-filling stage), May 21 (late-filling stage), and at maturity. The spikes with basically the same growth vigor were selected, and the spikes were divided into three parts: upper spike, middle spike, and basal spike; according to the number and position of the spikelet, each spike was divided into three sections (basal, middle, and upper). If the total number of spikelets was not a multiple of 3, the excess was included in the upper spike [33,35,69]. In each plot, 20 spikes were taken, of which 10 spikes were put into envelopes for preservation, dried, and ground to 100 mesh sieves for determination of soluble sugar content, free amino acid content, amylose content, amylopectin content, and protein components. The remaining 10 spikes were divided into the upper, middle, and basal parts and preserved in tinfoil and frozen in liquid nitrogen and then stored at −80 °C in a refrigerator for the determination of starch synthesis-related enzymes and nitrogen metabolism-related enzyme activities.

#### 4.2.1. Soluble Sugar Contents

The contents of soluble sugars in wheat grains were determined using anthrone colorimetric method [70,71].

About 0.1–0.3 g of sample was boiled in 5–10 mL deionized water for 30 min in 10 mL centrifuge tubes, then centrifuged at 4000× *g* for 5 min after cooling. The supernatants were decanted into 25 mL volumetric flasks. These steps were repeated twice and the supernatants were pooled. Then, 100 μL pooled supernatants were boiled with 3 mL anthrone reagent for 10 min, and the soluble sugar content was measured using a UV–Vis spectrophotometer (UV-2700, Daojin Instruments, Suzhou, China), which determined the absorbance at a 620 nm wavelength and calculated the soluble sugar content.

#### 4.2.2. Amylose and Amylopectin Contents

The amylose content was measured using the standard iodine colorimetry method. The amylopectin content is the difference between starch content and amylose content [18].

About 0.3 g of sample was added to 10 mL distilled water in a water bath at 100 °C for 60 min. The extract was centrifuged at 5000× *g* for 5 min and then the supernatant was poured away. The extraction was repeated three times. The remaining precipitate after repeated extraction was added with 2 mL distilled water to gelatinize in a water bath at 100 °C for 15 min. After cooling, the sample was mixed with 2 mL 9.2 mol L^−1^ HClO_4_ and intermittently vibrated for 15 min, followed by the addition of 2 mL 4.6 mol L^−1^ HClO_4_ and vibration for 15 min. After the extraction, 2 mL distilled water was added and the sample was centrifuged at 5000× *g* for 5 min. About 4 mL of the supernatant was taken and put into a 100 mL volumetric flask, and the volume was fixed with distilled water. Subsequently, 1 mL of the solution was taken to mix with 1 mL distilled water and 5 mL chromogenic reagent (2 g of anthrone dissolved in 1 L of concentrated sulfuric acid). Two minutes later, the absorbance was determined at 620 nm using a UV–Vis spectrophotometer (UV-2700, Daojin Instruments, Suzhou, China). The starch contents were calculated from a standard curve of different glucose concentrations.

The amylose content was measured using the standard iodine colorimetry method [18]. About 0.5 mL of anhydrous ethanol was added to a 0.25 g sample, and the suspension was thoroughly mixed. Then the sample was mixed with 4.5 mL 1 mol L^−1^ NaOH in a water bath at 100 °C for 10 min. After cooling, the dispersed sample was transferred to a 50 mL volumetric flask, and the volume was fixed with distilled water. About 1.25 mL of the supernatant was taken and put into a 25 mL volumetric flask, followed by the addition of 0.25 mL 1 mol L^−1^ CH_3_COOH, 0.5 mL KI-I_2_ (2 g KI and 0.2 g I_2_ in 0.1 L distilled water), and the volume was fixed with distilled water. The absorbance was measured at 620 nm on a UV–Vis spectrophotometer (UV-2700, Daojin Instruments, Suzhou, China) after 20 min. Determination of amylose content was performed according to a standard curve developed by using different ratios of amylose.

#### 4.2.3. Contents of Free Amino Acid (FAA)

Determination of free amino acid content was performed using the ninhydrin procedure [72,73]. Weigh 0.1 g sample into a 10 mL centrifuge tube, add 8 mL of 80% ethanol solution, and after mixing, add to water bath at 80 °C for 30 min. Centrifuge at 3000 r/min for 10 min, repeat extraction for three times, then fix the volume to 25 mL and mix well. Aspirate and add 2 mL of filtrate, 3 mL ninhydrin solution, and 0.1 mL 0.1% ascorbic acid solution, add to boiling water bath at 100 °C for 15 min, and cool to 5 mL with 60% ethanol. Content was determined colorimetrically at 620 nm using a UV–Vis spectrophotometer (UV-2700, Daojin Instruments, Suzhou, China).

#### 4.2.4. Determination of Related Enzymes in Grain Starch and Protein Synthesis

GS and GOGAT were determined using the method described by [22,23]. In total, 0.1 g of fresh wheat grain tissues were ground with 3 mL of extraction (pH 8, 0.05 mol L^−1^ Tris-HCl, 2 m mol L^−1^ MgSO_4_, 2 m mol L^−1^ DTT, and 0.4 mol L^−1^ sucrose) solution in an ice bath, followed by centrifugation for 10 min at 15,000× *g* at 4 °C. For GOGAT activity, the reaction was initiated by the addition of L-glutamine and NADH after enzyme preparation. A decrease in absorbance was recorded at 340 nm for 5 min. For GS activity, 150 µL of supernatant was added to 300 µL of reaction mixture, followed by incubation at 37 °C for 30 min. Finally, absorbance was measured at 540 nm using a UV–Vis spectrophotometer (UV-2700, Daojin Instruments, Suzhou, China).

SSS and GBSS were determined according to the method of [20,23]. In total, 0.1 g of fresh wheat grain tissues were ground with 1 mL of extraction solution (pH 7.5, 4 °C) containing 50 m mol L^−1^ Hepes-NaOH, 5 m mol L^−1^ EDTA, 1 m mol L^−1^ DTT, 2 m mol L^−1^ KCl, and 1% (*w*/*v*) PVP in an ice bath, followed by centrifugation for 5 min at 1000× *g* at 4 °C, and the resulting supernatant was used for the determination of SSS activities. The precipitate was resuspended in 0.5 mL extraction solution and used for the determination of GBSS activity. Content was determined colorimetrically at 340 nm using a UV–Vis spectrophotometer (UV-2700, Daojin Instruments, Suzhou, China).

#### 4.2.5. Grain Yield and Yield Components

At the wheat maturity stage, two 1 m rows were collected in each plot, and the spike numbers were measured. Thirty single stems were collected randomly in each plot to record the kernel number per spike. A 2 m × 2 m (4 m^2^) area of wheat in each plot was harvested manually (avoiding border rows), subjected to threshing, and weighed post-natural drying to measure the yield of the respective plot. The grain yield at corresponding moisture contents was recorded and expressed against a standard moisture content of 13%. Grain moisture content was determined using a grain moisture meter (PM-8188 New, Beijing Hexinchang Science and Technology Development Co., Ltd. Beijing, China).

#### 4.2.6. Contents of Protein Components

Extraction of protein components was determined as described by Yao et al. [74]. In total, 0.5 g of grain sample was mixed with 5 mL distilled water and extracted for 30 min on a 180 rpm shaking table at 25 °C. After centrifugation at 4000 rpm for 30 min, the supernatant was collected for determining albumin. The residue was extracted with 5 mL of 2% NaCl for 30 min on a 180 rpm shaking table at 25 °C. The homogenate was centrifuged at 4000 rpm for 30 min to obtain globulins. The residue was blended with 75% ethanol and shocked in an 80 °C water bath for 5 min and at 25 °C for 15 min, then centrifuged at 4000 rpm for 30 min to extract the gliadin. Finally, the residue was extracted with 0.2% NaOH at 25 °C for 30 min. After centrifuging at 4000 rpm for 30 min, glutenin was obtained. Each extraction was repeated three times. The four protein extracts and the residual parts were poured into a dissolving tube and the protein concentration was determined by Kjeldahl nitrogen determination method [75]. Grain protein concentration was calculated as grain N concentration × 5.7 (Reference National Standard of the People’s Republic of China for conversion coefficient) [76].

#### 4.2.7. Determination of Soil Physical and Chemical Properties

Soil samples were collected from the 0 to 20 cm tillage layer, air-dried, and sieved. Total soil N was measured using the Kjeldahl method [77]. Briefly, 1 g soil sample into a Kjeldahl flask. Add 1.1 g of catalyst (K_2_SO_4_ to CuSO_4_•5H_2_O ratio, 10:1) and 3 mL of concentrated sulfuric acid. Fit the flask with a funnel and digest the mixture. After cooling, put it into Kjeldahl nitrogen analyzer for determination (Hanon Future Technology Group Co., Ltd., Jinan, China, Hanon Instruments K9840).

Take 5 g of soil sample into a 250 mL conical flask. Add 100 mL of 0.5 mol L^−1^ Na-HCO_3_, shake for 30 min. Filter the mixture and determine available phosphorus content by sodium hydrogen carbonate solution–Mo–Sb anti spectrophotometric method [77].

Take 5 g of soil sample into a 250 mL conical flask. Add 50 mL of 1 mol L^−1^ NH_4_OAc, shake for 30 min. Filter the mixture and determine available potassium content by flame photometer [77].

The soil pH was determined in a 1:2.5 (soil-to-water ratio) suspension using a glass electrode [77]. We measured SOC using the external heating potassium di-chromate oxidation method [77,78]. Briefly, 0.1 g soil sample was transferred to a hard glass tube and then 5 mL 0.8 mol L^−1^ K_2_Cr_2_O_7_ and 5 mL H_2_SO_4_ was added. The samples were heated at 180 °C in an oil bath until boiled for 5 min and then titrated with 0.2 mol L^−1^ FeSO_4_.

### 4.3. Statistical Analysis

The data were processed using Microsoft Excel 2013 (Redmond, WA, USA) and statistical analysis. ANOVA mean comparisons were performed in terms of the least significant difference (LSD), at the significance level of *p* < 0.05. The data were fitted, and figures were generated using Origin8.5 (Origin Lab, Northampton, MA, USA).

## 5. Conclusions

This study evaluated the effects of nitrogen topdressing on the physiological and biochemical characteristics of grains at upper, middle, and basal spike positions. Moderate nitrogen application (75–112.5 kg ha^−1^) significantly increased soluble sugar content, enhanced GBSS and SSS activities, and promoted amylose and amylopectin accumulation, whereas excessive nitrogen (150 kg ha^−1^) had inhibitory effects. Upper and basal spikelets responded more strongly, with higher GS and GOGAT activities, increased free amino acid content, and enhanced protein accumulation, particularly glutenin, effectively reducing disparities among spike positions. These results indicate that optimal nitrogen topdressing facilitates assimilate transport and synergistically regulates key enzymes of carbon and nitrogen metabolism, ultimately improving both grain yield and quality.

## Figures and Tables

**Table 1:** Effects of different nitrogen topdressing rates on wheat yield and yield components.

Treatment	Spike Number (×10^4^ ha^−1^)	Grain Number per Spike	1000-Grain Weight (g)	Grain Yield (kg ha^−1^)
N1	459.83 d	38.76 b	48.71 c	7357.46 c
N1 + 37.5	483.05 cd	40.26 b	50.04 b	8031.04 b
N1 + 75	564.96 b	44.81 a	50.71 ab	9206.64 a
N1 + 112.5	630.57 a	45.05 a	51.53 a	9451.74 a
N1 + 150	506.28 c	42.69 ab	47.58 c	8076.03 b

Different lowercase letters in the same group indicate significant differences among treatments at *p* < 0.05. Abbreviations: N1—Basal nitrogen application of 150 kg ha^−1^; N1 + 37, N1 + 75, N1 + 112.5, N1 + 150—Based on the basal application of nitrogen fertilizer 150 kg ha^−1^ and topdressing at the jointing stage of 37.5, 75, 112.5, and 150 kg ha^−1^, respectively.

## Data Availability

The original contributions presented in this study are included in the article. Further inquiries can be directed to the corresponding authors.
